# Analysis of a cancer-associated mutation in the budding yeast Nuf2 kinetochore protein

**DOI:** 10.17912/micropub.biology.001546

**Published:** 2025-03-11

**Authors:** Angelica Andrade Latino, Sue Biggins

**Affiliations:** 1 Division of Basic Sciences, Fred Hutch Cancer Center, Seattle, Washington, United States; 2 Howard Hughes Medical Institute

## Abstract

The kinetochore is a highly conserved megadalton protein complex that ensures proper chromosome segregation via microtubule attachments. The NDC80 complex is one of the major conserved microtubule binding complexes in the kinetochore. NUF2, a protein within the NDC80 complex, has been identified as a cancer gene candidate because missense mutations, found across different tumor samples, cluster within NUF2’s calponin homology domain. In this study, we examined a NUF2 cancer-associated mutation in a simple and well-studied organism, *Saccharomyces cerevisiae* , to elucidate its effects on cell division. We studied the budding yeast *nuf2 ^Q21A^* mutation with the intention of extrapolating our results to the homologous cancer associated mutation in *Homo sapiens* NUF2 ^R19H^ (HsNUF2 ^R19H^ ). Our studies demonstrate that the *nuf2 ^Q21A^* mutant does not exhibit any growth defects or disrupt kinetochore composition. Additionally, it does not affect the Ndc80 complex’s interactions with the Dam1 complex or with the Mps1 kinase. These results indicate that the yeast *nuf2 ^Q21A^* mutant does not cause a significant defect in kinetochore function, and that the role of HsNUF2 ^R19H^ in cancer will need to be further investigated by directly studying the cancer-associated mutation in human cells.

**Figure 1. Characterization of the S. cerevisiae nuf2 Q21A mutant:**
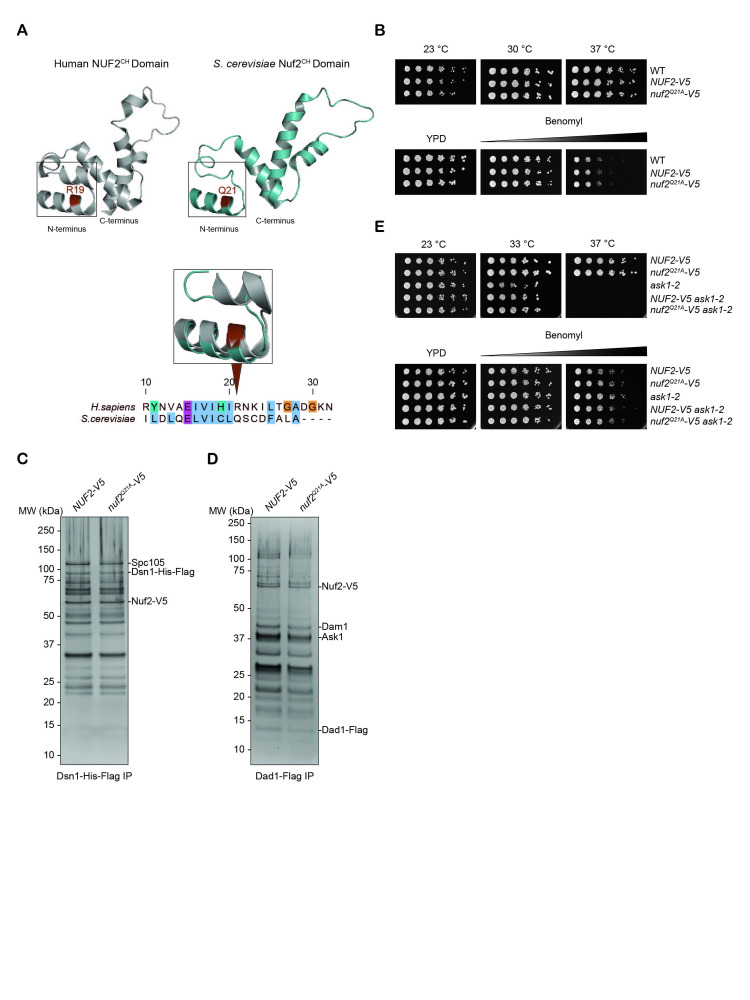
(A) Top: Side-by-side protein structure comparison of the human (in gray; PDB: 2VE7) and *S. cerevisiae* (in deep teal; PDB: 5TCS) Nuf2 ^CH^ domains. Structurally conserved residues HsNUF2-R19 and Nuf2-Q21 are highlighted in brown, and both are situated in the same alpha helix of the protein. Inset: Protein structure alignment of amino acids 10-30 of HsNUF2 and amino acids 12-32 of *S. cerevisiae* Nuf2 *.* Conserved residues highlighted in brown. Bottom: Jalview Nuf2 protein sequence alignment of the amino acids displayed in the inset. Clustal color scheme denotes colored residues as conserved, and the different colors indicate the biochemical properties of the side chains. (B) Five-fold serial dilution assay of WT (SBY4), *NUF2-V5* (SBY22754), and *nuf2 ^Q21A^ -V5* (SBY22924) strains to test temperature and benomyl sensitivity. Strains were grown for 2-3 days on YPD at the indicated temperatures and on different concentrations of benomyl (5 μg/ml and 15 μg/ml). (C) Flag immunoprecipitation of Dsn1-His-Flag from *NUF2* - *V5* (SBY23066), and *nuf2 ^Q21A^ -V5* (SBY23028) genetic backgrounds analyzed via silver-stained SDS-PAGE. (D) Flag immunoprecipitation of Dad1-Flag from *NUF2* - *V5* (SBY23067) and *nuf2 ^Q21A^ -V5* (SBY23029) genetic backgrounds were analyzed via silver-stained SDS-PAGE. (E) Five-fold serial dilution assay tested *NUF2-V5* (SBY23133), *nuf2 ^Q21A^ -V5* (SBY23073), *ask1-2* (SBY1300), *NUF2-V5* ask1-2 (SBY23192), and *nuf2 ^Q21A^ -V5 ask1-2* (SBY23190) for temperature and benomyl sensitivity. Strains were grown for 2-3 days on YPD at the indicated temperatures and on different concentrations of benomyl (5 μg/ml and 15 μg/ml).

## Description

All eukaryotic cells undergo mitosis, a key event required to produce two genetically identical daughter cells during cell division (Santaguida and Musacchio 2009). Defects in this crucial process can lead to chromosome mis-segregation events that result in aneuploid cells, a hallmark of cancer (Holland and Cleveland 2009). Proper chromosome segregation during mitosis requires the kinetochore, a highly conserved and tightly regulated protein network that assembles at centromeres and forms microtubule attachments (Santaguida and Musacchio 2009; Musacchio and Desai 2017; Ariyoshi and Fukagawa 2023). During anaphase, microtubules pull chromosomes to opposite poles of a dividing cell via kinetochore attachments to ensure proper inheritance of the genetic material.

The two major budding yeast kinetochore subcomplexes responsible for forming microtubule attachments are the Ndc80 and Dam1 complexes (Ndc80c and Dam1c; Biggins 2013). The role of the Ndc80c is to form stable, load-bearing attachments to dynamic microtubules. The Dam1c facilitates these attachments by interacting with the Ndc80c and oligomerizing around microtubules enabling the Ndc80c to track the tip of dynamic microtubules with higher affinity (Westermann et al., 2005; Westermann et al., 2006; Lampert et al., 2010; Tien et al., 2010; Lampert et al., 2013). The highly conserved Ndc80c is a heterotetramer comprised of Ndc80p-Nuf2 and Spc24-Spc25 heterodimers (Wei et al., 2005). Each heterodimer is positioned on opposite ends of the dumbbell shaped complex with Ndc80p-Nuf2 oriented to interact with the microtubule while Spc24-Spc25 connects the complex to the rest of the kinetochore (Wei et al., 2005; Cheeseman et al., 2006). The N-terminus of each protein within the Ndc80p-Nuf2 heterodimer folds into independent globular calponin homology (CH) domains (Wei et al., 2007; Ciferri et al., 2008). The CH domain of Ndc80p interacts with microtubules directly, whereas the CH domain of Nuf2 does not (Alushin et al., 2010; Sundin et al., 2011). However, the Nuf2 ^CH^ domain plays a critical role in chromosome segregation. In *S. cerevisiae* , the Nuf2 ^CH^ domain contains a conserved patch that is a docking site for the Dam1c and the kinase Mps1 that regulates kinetochore-microtubule attachments (Maure et al., 2007; Sarangapani et al., 2021; Parnell et al., 2024; Pleuger et al., 2024; Zahm et al., 2024). In HeLa cells, charge reversal mutations within the NUF2 ^CH^ domain resulted in cells arresting in mitosis (Sundin et al., 2011).

A previous study mapped somatic missense mutations, identified from different cancerous tumor samples, onto the available PDB (2VE7) structure of NUF2 and found that they significantly cluster within its CH domain (Kamburov et al., 2015). We speculated that the NUF2 cancer associated missense mutations could affect the structure of the CH domain and alter its protein function within cancerous cells. The aim of this study was to elucidate the effects that the human cancer associated missense mutations in NUF2 have on kinetochore composition and function by examining them in *S. cerevisiae* . Since the mutants are viable in cancer cells, we used budding yeast as a tractable system because it provides assays that allow for more in-depth analyses of these mutations than in human cells. For example, we can perform sensitive growth assays as well as study genetic and biochemical interactions between kinetochore proteins to uncover the functional consequences of the cancer-associated mutations.

The CH domain of NUF2 is conserved across *H. sapiens* and *S. cerevisiae* (Ciferri et al., 2008), so we performed a structural alignment of both proteins to identify the location of the cancer-associated residues in the yeast protein. Utilizing PyMOL, we aligned the NUF2 protein structures from the *H. sapiens* NDC80c ^Bonsai^ and *S. cerevisiae* Ndc80c ^Dwarf^ PDBs ( Fig. 1A, top). Out of the 8 cancer-associated residues identified (Kamburov et al., 2015), 6 are structurally conserved in *S. cerevisiae* . Out of the 6 structurally conserved residues, we focused on the HsNUF2-R19 amino acid since it has the highest mutation frequency ( cBioPortal ) and is equivalent to Nuf2-Q21 in *S. cerevisiae* as depicted by the overlay of the two protein structures ( Fig. 1A, inset). A recent structural study revealed the Nuf2-Q21 residue to be nestled within an interacting pocket between the Ndc80c ^CH^ domains and the Dam1c (Muir et al., 2023; Zahm et al., 2023). Residues of the Dam1c that are involved in this interaction are phosphoregulated by Aurora B/Ipl1 kinase when erroneous kinetochore-microtubule attachments are formed (Keating et al., 2009). The Nuf2-Q21 residue also lies near a highly conserved “interaction hub” in the Nuf2 ^CH^ domain that binds to Mps1 and the Dam1c (Parnell et al., 2024; Pleuger et al., 2024; Zahm et al., 2024). Therefore, a mutation at the Nuf2-Q21 amino acid could alter these vital interactions.

The Nuf2-Q21 residue is positioned on the outer surface of an alpha helix, most likely engaging in intermolecular interactions with other nearby proteins. We therefore generated a mutant that would most effectively disrupt interactions by substituting the glutamine with an alanine to introduce a cavity. The *nuf2 ^Q21A^* mutant was made in a *NUF2-V5* background strain at the endogenous locus via CRISPR/Cas9 mutagenesis.

To test for growth defects, we performed a serial dilution growth assay. Strains were plated at various temperatures or onto various concentrations of benomyl, a microtubule depolymerizing agent ( Fig. 1B ). The assays demonstrated that the presence of the V5 epitope tag does not affect cell viability and that the *nuf2 ^Q21A^* mutant did not exhibit growth defects under any of the tested conditions.

We next examined kinetochore composition by purifying the Dsn1 protein because it isolates most of the budding yeast native kinetochore (Akiyoshi et al., 2010; Gupta et al., 2018). We purified Dsn1 from WT and *nuf2 ^Q21A^* mutant cells and analyzed kinetochore composition through silver-stained SDS-PAGE analysis. The *nuf2 ^Q21A^* mutant had no detectable changes in kinetochore composition compared to WT ( Fig. 1C ). Some kinetochore proteins that might be affected by the presence of the *nuf2 ^Q21A^* , such as the Mps1 kinase, are not easily detected via silver-stained analysis. As such, we performed mass spectrometry (MS) on the Dsn1-His-Flag immunoprecipitation samples. MS analysis revealed Mps1 to be among the most enriched proteins detected; however, there were no significant changes in its abundance between WT and the *nuf2 ^Q21A^* mutant (Extended data files 1 and 2). Additionally, the MS analysis further corroborated the findings of the silver-stained analysis in that there were no significant changes in peptide counts detected across the co-precipitated kinetochore proteins (Extended data files 1 and 2). Together, these findings indicate the *nuf2 ^Q21A^* mutant does not perturb cell viability, or the Ndc80c’s interaction with Mps1 kinase and other kinetochore proteins isolated in the Dsn1-His-Flag purification.

Next, we tested whether the *nuf2 ^Q21A^* mutant affected physical or genetic interactions between the Ndc80c and the Dam1c. Since the Dam1c is substoichiometric in the Dsn1-His-Flag purifications, we directly purified the Dam1c component Dad1 via a Dad1-Flag co-immunoprecipitation experiment to assay its interaction with Nuf2 ( Fig. 1D ). In both WT and the mutant, similar amounts of Nuf2 co-purified with Dad1, indicating that the *nuf2 ^Q21A^* mutant does not disrupt interactions between the Ndc80c and the Dam1c. We also asked whether the *nuf2 ^Q21A^* mutant exhibited genetic interactions with a mutant in the Dam1c. We introduced the *nuf2 ^Q21A^* mutant into a background strain that cripples the Dam1c using the temperature sensitive allele *ask1-2* (Li et al., 2002). The double mutant was viable even when its microtubule attachments were challenged and did not exhibit temperature sensitivity at the *ask1-2* semi-permissive temperature of 33 °C ( Fig. 1E ). Taken together, these data suggest that the *nuf2 ^Q21A^* mutant does not have a major effect on Ndc80c function or affect the Ndc80c’s interaction with the Dam1c.

In sum, we demonstrated that the *nuf2 ^Q21A^* mutant does not have detectable effects on kinetochore composition or exhibit any growth defects, suggesting it does not influence kinetochore-microtubule attachments. It is therefore unclear whether the homologous HsNUF2 ^R19H^ missense mutation contributes a significant role in the tumorigenesis of the cancers it was found in. It is important to note that the HsNUF2 ^R19H^ is not a hotspot mutation and was obtained from an outdated dataset. With the emergence of new sequencing profiles of more tumor samples, new mutations should be considered for future studies. For example, the HsNUF2 ^S340L^ now has a higher mutation frequency than the HsNUF2 ^R19H^ ( cBioPortal ). Ultimately, the HsNUF2 ^R19H^ mutant should be studied in human cells as the region this residue occupies is not highly conserved at the primary sequence ( Fig. 1A ). Consistent with this limitation, we were unable to generate a chemically similar HsNUF2 ^R19H^ mutation in yeast since the chemical properties of the arginine and glutamine side chains are distinctly different from one another. Additionally, the Dam1c complex is not conserved but has a functional ortholog called the SKA complex in metazoan cells (Hanisch et al., 2006; Welburn et al., 2009). Cross-linking mass spectrometry demonstrates a potential interaction between the CH domains of the mammalian NDC80c with the C-terminus of SKA3, a protein within the SKA complex (Helgeson et al., 2018). It will therefore be interesting to explore whether HsNUF2 ^R19H^ mutation affects the NUF2-SKA3 interaction in human cells. Similarly, although the interaction between MPS1 and NUF2 ^CH^ domain is conserved in metazoan cells, the region at which the interaction occurs differs. In human cells, the NUF2 ^CH^ domain contacts the middle region of the MPS1 kinase (Ji et al., 2015) whereas in *S. cerevisiae* the interaction occurs at the N-terminal tail of Mps1 (Parnell et al., 2024; Pleuger et al., 2024; Zahm et al., 2024). Hence the different modes of interaction may account for the lack of phenotype observed in the *nuf2 ^Q21A^* mutant. In conclusion, future studies in human cells may shed light on the function of HsNUF2-R19 residue and its potential role in cancer.

## Methods

Culture conditions

All *S. cerevisiae* strains used in this study were grown in yeast extract peptone dextrose (YPD) liquid cultures, containing 2% D-glucose (Sigma-Aldrich) and 0.02% adenine (MP Biomedicals). Cultures were grown at 23-30 ^°^ C and were harvested at late-log phase OD _600nm_ = ~3.

Spotting dilution assay

Strains were grown overnight in 5 ml of YPD liquid. Utilizing the SmartSpec Plus spectrometer (Bio-Rad), the concentration of cells was measured at OD _600nm_ and were diluted to OD _600nm_ = 1. Each strain underwent a five-fold serial dilution in a 96-well plate. The first column of the plate contained fully saturated OD _600nm_ = 1 cells and subsequent wells had a 1:5 dilution in water. Strains were spotted onto YPD and YPD benomyl coated plates (5 μg/ml and 15 μg/ml). YPD plates containing benomyl were incubated at 23 ^°^ C while the plates containing just YPD were incubated at the indicated temperatures. Cells were grown for 3-5 days.

One-step purifications & silver-staining

Flag purifications were performed as previously described (Gupta et al., 2018) except lysates were treated with benzonase as described below. Briefly, cells were harvested during late-log phase (OD _600nm_ = ~3) and then flash frozen in Buffer H/0.15 (25 mM HEPES pH 8.0, 2 mM MgCl _2_ , 0.1 mM EDTA pH 8.0, 0.5 mM EGTA-KOH pH 8.0, 15% Glycerol, 0.1% NP-40, and 150 mM KCl) containing phosphatase inhibitors (1 mM sodium-pyrophosphate, 2 mM sodium-β-glycerophosphate, 0.1 mM sodium orthovanadate, and 5 mM NaF), 0.1 mM microcystin-LR, 0.2 mM PMSF, and protease inhibitors (10 μg/mL for each of the following leupeptin, pepstatin A, and chymostatin). Pellets were lysed via a Freezer Mill (SPEX SamplePrep) and were then treated with 50 units/ml of benzonase (EMD Milipore Corp) for 30 minutes on ice. Treated lysates were then ultracentrifuged at 24,000 RPM for 90 minutes at 4 ^°^ C and protein layer was extracted. The Pierce BCA Protein Assay Kit (Thermo Scientific) was used to measure the protein concentration within each extract which were subsequently normalized for each immunoprecipitation experiment. Conjugated Flag Protein G Dynabeads were made via crosslinking the α-M3DK antibody (Barrero et al., 2024) [GenScript] to Protein G Dynabeads (Invitrogen). These beads were incubated with the normalized protein extracts for 3 hours at 4 °C. After the incubation, beads were washed three times with Buffer H/0.15 containing phosphatase inhibitors, protease inhibitors, 0.1 mM microcystin-LR, 2 mM DTT and then two times with Buffer H/0.15 containing protease inhibitors, and LPC. Proteins were eluted off beads into Buffer H/0.15 containing 0.2 mM PMSF, protease inhibitor stock and 0.5mg/mL 3 x M3DK Peptide (Sigma-Aldrich) via gentle agitation for 30 minutes at room temperature.

5-10 μl of the immunoprecipitated samples were boiled in 1x sample buffer (Invitrogen 1x LDS sample buffer, 5% β-mercaptoethanol, and sterile water) for 5 mins at 95 °C. Each immunoprecipitated sample was run on precast 4-12% Bis-Tris gels (Thermo Fisher Scientific). Gels were stained with silver-staining agents following the Silver Quest Staining Kit (Invitrogen) instructions. The ChemiDoc MP system (Bio-Rad) was used for imaging.

Protein sequence alignment

Nuf2 protein sequences were obtained from UniProt ( *Homo sapiens,* Q9BZD4) and the *Saccharomyces* Genome Database (W303 background). Entire protein sequence alignment was generated utilizing the default conditions of the Clustal Omega algorithm in Jalview (version 2.11.4.1). Clustal color scheme was used to denote the conservation and the biological properties of the side chains. Image was exported as an EPS file.

Protein structure alignment

Utilizing PyMOL (version 3.1.3), Nuf2 ^CH^ domains from the following PDB structures were isolated: PDB: 2VE7 (NDC80c ^Bonsai^ ) and PDB: 5TCS (Ndc80c ^Dwarf^ ). Alignment was performed of only amino acids 10 or 12 to 100 of each Nuf2 ^CH^ domain. Conserved residues were identified through the alignment. Protein structures were then individually exported as PNGs in the aligned orientation. A similar procedure was followed for the inset, except only residues 10-30 of HsNUF2 and residues 12-32 of *Saccharomyces cerevisiae* Nuf2 were aligned.

Mass spectrometry preparation and analysis

Immunoprecipitated samples were prepared as indicated under the one-step purification method until the last wash step. After the last wash, samples were rinsed two times with pre-elution rinse buffer (50 mM Tris pH 8.3, 75 mM KCl, and 1 mM EGTA). The samples were eluted via gentle agitation on a Vortex-Genie 2 (Scientific Industries) set to the lowest setting for 30 minutes at room temperature. Each sample was eluted in 70 μl of 0.2% RapiGest (Waters Corporation) in 50 mM ammonium bicarbonate. 10 μl of each elution sample was used for silver-stained analysis as indicated under silver-staining methods while the remaining 60 μl was sent for mass spectrometry processing.

Samples were prepared for LC-MS/MS analysis by reducing disulfide bonds, capping free cysteines, and digesting the proteins with trypsin. The resulting peptide samples were analyzed by LC-MS/MS on an Thermo Eclipse mass spectrometer. The mass spectrometry data collected in this study is available through Mass Spectrometry Interactive Virtual Environment (MassIVE, University of California San Diego; ftp://massive.ucsd.edu/v09/MSV000097121/ ).

## Reagents

Yeast strains and plasmid methods

To generate the *NUF2-3V5:HIS3Mx6* strain, a *3V5:HIS3Mx6* cassette was amplified from pSB3158 using PCR. This cassette was then inserted into the endogenous locus of *NUF2* at the 3’ of the coding region replacing the stop codon via homologous recombination (Petracek and Longtine 2002). To generate the *nuf2 ^Q21A^* missense mutation in this strain, a single-step CRISPR/Cas9 edit was performed (Akhmetov et al., 2018). *DAD1-3Flag* was made as described (De Regt et al., 2022). *DSN1-6His-Flag* was generated as described (Akiyoshi et al., 2010). The *ask1-2* yeast strain was a gift from Stephen Elledge’s laboratory (Harvard University, Cambridge, MA) and plasmids pSB3158 and pSB3218 were gifts from Elçin Ünal’s Laboratory (University of California, Berkeley, CA).

*Saccharomyces cerevisiae* strains used throughout this study are derivatives of SBY3 and SBY4 (W303).

**Table:** 

Strains	Genotype	Reference
SBY3 (W303)	Mat **a** *ura3-1 leu2-3,112 his3-11 trp1-1 can1-100 ade2-1 bar1-1*	Biggins Lab
SBY4 (W303)	Matα *ura3-1 leu2-3, 112 his3-11 trp1-1 can1-100 ade2-1 bar1-1*	Biggins Lab
SBY12465	Matα *DAD1-3Flag:TRP1* *LYS2*	De Regt et al., 2022
SBY1300	Mat **a** *ask1-2*	Gift from Elledge’s Laboratory, Harvard University, Cambridge, MA
SBY22754	Matα *NUF2-3V5:HIS3Mx6*	This study
SBY22924	Matα *nuf2 ^Q21A^ -3V5:HIS3Mx6 *	This study
SBY23028	Mat **a** *nuf2 ^Q21A^ -3V5:HIS3Mx6 DSN1-6His-3Flag:URA3 *	This study
SBY23029	Matα *nuf2 ^Q21A^ -3V5:HIS3Mx6 DAD1-3Flag:TRP1 *	This study
SBY23066	Mat **a** *NUF2-3V5:HIS3Mx6 DSN1-6His-Flag:URA3*	This study
SBY23067	Matα *NUF2-3V5:HIS3Mx6 DAD1-3Flag:TRP1*	This study
SBY23073	Mat **a** *nuf2 ^Q21A^ -3V5:HIS3Mx6 *	This study
SBY23133	Mat **a** *NUF2-3V5:HIS3Mx6*	This study
SBY23190	Mat **a** *nuf2 ^Q21A^ -3V5:HIS3Mx6 ask1-2 *	This study
SBY23192	Mat **a** *NUF2-3V5:HIS3Mx6 ask1-2*	This study

Plasmids used in this study.

**Table:** 

Plasmid number	Purpose	Reference
pSB3158	Integration of the *3V5:HIS3Mx6* cassette at the endogenous *NUF2* locus.	Gift from Elçin Ünal’s Laboratory, University of California, Berkeley, CA Janke et al., 2004
pSB3218	Generate sgRNA used for CRISPR/Cas9 mutagenesis.	Gift from Elçin Ünal’s Laboratory, University of California, Berkeley, CA
pSB3608	Generated sgRNA (pSB3218 derivative) used for Nuf2-Q21 CRISPR/Cas9 mutagenesis.	This study

Oligonucleotides used in this study.

**Table:** 

Oligo number	Purpose	Sequence	Reference
SB8342	Forward oligo for Gibson assembly to generate sgRNA for *nuf2 ^Q21A^* mutagenesis. Contains homology to pSB3218.	5’- ggctgggcaacaccttcgggtggcgaatggGTTCCCCATTTTGGATCTAC -3’	This study
SB8343	Reverse oligo for Gibson assembly to generate sgRNA for *nuf2 ^Q21A^* mutagenesis. Contains homology to pSB3218.	5’- attttaacttgctatttctagctctaaaacGTAGATCCAAAATGGGGAAC -3’	This study
SB8350	Forward primer to amplify *3V5:HIS3Mx6* from pSB3158 containing homology to *NUF2* . Used for PCR tagging.	5’- TATTAATAAATACATGAATGAAATGCTCGAATATATGCAAgcggccgctctagaactagt -3’	This study
SB8351	Reverse primer to amplify *3V5:HIS3Mx6* from pSB3158 containing homology to *NUF2* . Used for PCR tagging.	5’- AGAAAACACAGAAGGGGGAGTAAAAATAAGTATACCGCTGccccctcgaggtcgacggta -3’	This study
SB8371	Forward primer to amplify entire *NUF2* coding region.	5’- CTCCAGCATCCCTTGAGCAA -3’	This study
SB8372	Reverse primer to amplify entire *NUF2* coding region.	5’- ACCTTCACATGTTCCGCAGA -3’	This study
SB8373	Repair template for *nuf2 ^Q21A^* mutagenesis via CRISPR.	5’- GAAAAAATACTGGTAAAAAGCATGTACTGAGGAGAAAGGCTCCAGCATCCCTTGAGCAAAATGAGTAGGAATCAAGATGTGTTCCCCATTTTGGATCTACAAGAACTAGTTATATGTTTGCATAGCTGTGATTTTGCGCTAGCCACACAGGAAAATATCTCTAGGCCCACCTCAGACTACATGGTAACCCTTTACAAACA -3’	This study
SB8466	Amplify 5' end of *NUF2* coding region.	5’- TGCGGTTAAGTCGTCTAACG -3’	This study

Antibody used in this study:

**Table:** 

Name	Type	Species	Brand	Reference
M3DK (α-Flag)	Monoclonal	Mouse	GenScript	(Barrero et al., 2024)

## Data Availability

Description: Description: Mass spectrometry analysis of Dsn1-His-Flag purifications of WT. Resource Type: Dataset. DOI: https://doi.org/10.22002/hr7nz-4mb27 Description: Mass spectrometry analysis of Dsn1-His-Flag purifications of nuf2-Q21A-V5. Resource Type: Dataset. DOI: https://doi.org/10.22002/kvpk3-pt771
